# Mechanism of T cell regulation by microRNAs

**DOI:** 10.7497/j.issn.2095-3941.2013.03.002

**Published:** 2013-09

**Authors:** Juan Liu, Chang-Ping Wu, Bin-Feng Lu, Jing-Ting Jiang

**Affiliations:** 1 Department of Tumor Biological Treatment, The Third Affiliated Hospital of Soochow University, Changzhou 213003, China;; 2 Department of Immunology, University of Pittsburgh School of Medicine, Pittsburgh, PA 15261, USA

**Keywords:** MicroRNA, T cell, gene expression

## Abstract

MicroRNAs (miRNAs) are small, non-coding single-stranded RNAs that can modulate target gene expression at post-transcriptional level and participate in cell proliferation, differentiation, and apoptosis. T cells have important functions in acquired immune response; miRNAs regulate this immune response by targeting the mRNAs of genes involved in T cell development, proliferation, differentiation, and function. For instance, miR-181 family members function in progression by targeting Bcl2 and CD69, among others. MiR-17 to miR-92 clusters function by binding to CREB1, PTEN, and Bim. Considering that the suppression of T cell-mediated immune responses against tumor cells is involved in cancer progression, we should investigate the mechanism by which miRNA regulates T cells to develop new approaches for cancer treatment.

## Introduction

Cancer progression is associated with many gene mutations and abnormal gene expressions. As a result, tumor-specific and tumor-associated antigens, which can stimulate T cell immune responses against malignant cells, are produced^1^. Immune responses to tumors consist of three stages, namely, elimination, equilibrium, and escape^2^. In the elimination stage, cancer cells are recognized and eradicated by the immune system. If the immune system fails to eradicate tumor cells, the equilibrium stage is reached. In the equilibrium stage, tumor cells and immune cells coexist. In the escape stage, tumors grow by developing immune suppressive mechanisms to escape immune attack mediated primarily by type 1 immune cells. Hence, new approaches should be developed to fight cancer by investigated the exact mechanism by which microRNAs regulate the functions of T cells^3^^,^^4^.

Non-coding RNAs are indirectly involved in translation, particularly in regulating protein synthesis. These RNAs have several types, including small RNAs and long non-coding RNAs (lncRNAs). Similar to mRNA, lncRNAs are more than 200 nucleotides (nt) in length with a 5’ methyl cap and a polyA tail. However, small RNAs are less than 200 nt; these small RNAs include tRNA, rRNA, snRNA, siRNA, and microRNA (miRNA). MiRNAs are an important class of endogenously expressed small non-coding RNAs. A functional mRNA-targeting mature miRNA is single stranded and typically 19nt to 22nt in length after this type of miRNA is synthesized from double-stranded RNA^5^^-^^7^; mature miRNA also exhibits critical regulatory functions by modulating the rate of protein synthesis in eukaryotes^8^.

MiRNAs are initially transcribed by RNA polymerase II using a specific genomic DNA as template in cell nuclei. In this process, primary miRNA (pri-miRNA) sequences are produced, in which a hairpin sequence containing the mature miRNA is found. The hairpin of the pri-miRNA was further transformed into precursor miRNAs by the enzyme Drosha and then exported into the cytoplasm via Exportin V. Pre-miRNAs are cleaved into two individual strands of RNA by the enzyme Dicer. One strand of the miRNA is usually degraded and the other strand is associated with RNA-induced silencing complexes (RISC)^9^.

MiRNA molecules regulate gene expression mainly by binding to complementary sequences in the 3′-untranslated region (UTR) of target mRNAs and then integrating into RISC to suppress translation or to degrade miRNA-bound mRNA transcripts^10^^,^^11^. The functional significance of miRNAs is initially demonstrated in the developmental process of *Caenorhabditis elegans*^12^. Further studies have provided a strong link between miRNA and immune system function.

T cells are derived from lymphoid stem cells in the bone marrow and mature in the thymus. Based on the expression of surface molecules, such as CD3, CD4, and CD8, the development of T cells in thymus is divided into three stages: initial stage, in which T cells are formed as double negative cells (CD4^–^CD8^–^); intermediate stage, in which T cells are developed as double positive cells (CD4^+^CD8^+^); and final stage, in which T cells are formed as single positive cells (CD4^–^CD8^+^ or CD4^+^CD8^–^)^13^. Based on maturation status, mature T cells are divided into naive cells, effector cells, memory T cells, and exhausted/anergic cells; each subset expresses specific surface molecules, including CCR7, CD45RA, CD70, and CD27^14^^,^^15^. Based on cytokine profiles, T helper cells (Th) can be divided into Th1, Th2, Th17, and Th9 cells, which enhance the activity of other immune cells by producing various cytokines^16^^,^^17^. T cells function in several processes, mainly in recognizing allergens and secreting cytokines that eradicate foreign molecules and cancer cells. For example, CD8^+^T cell secretes granzyme and perforin that directly kill cancer cells. CD4^+^T helps other T cells function properly and memory T cells participate in secondary immune response.

T lymphocytes (T cells) are mainly involved in adaptive immune response; miRNA is involved in the regulation of T cell development, maturation, differentiation, and function^18^^,^^19^. In this review, recent findings and current understanding of the function of miRNAs in T cells are presented. Further studies should be conducted to elucidate the mechanism by which miRNAs regulate T cells in the context of immune therapy for malignant tumors and cancer immunosuppressive environments.

## MiRNAs and T cell development

miRNAs exhibit dynamic changes during the development of hematopoietic stem cells. The expression level of miRNAs is highly related to the degree of T cell differentiation and development^20^. For example, miR-125b is expressed at a higher extent in lymphoid stem cells than in myeloid stem cells and hematopoietic stem cells. This high expression stimulates the development of lymphocyte lineage. MiR-125b is also involved in hematopoietic stem cell survival and functions in the maintenance of lymphoid balance^21^. In hematopoietic progenitor cells, high miR-181a expressions facilitates T and B cell development^8^^,^^22^.

Evidence has shown that miRNA is involved in T cell development in the thymus. Dicer, an RNaseIII-like enzyme necessary to generate short interfering RNAs and miRNAs, is required for CD8 T cell development^23^^,^^24^. MiRNA expression is dynamically regulated in distinct stages of thymic T cell development^19^; this result suggests that miRNAs participate in the regulation of T cell differentiation in the thymus. MiR-181a, which is specifically enriched at the CD4^+^CD8^+^ DP stage of thymocyte development, suppresses the expression of Bcl-2, CD69, and T cell receptor (TCR); all of these molecules are important in positive selection. MiR-181a has also been shown to increase sensitivity to peptide antigens by downregulating multiple phosphatases^25^. These findings have indicated that miR-181a functions as an intrinsic “rheostat” in TCR signaling, which is very important in T cell development^26^. In addition to miR-181a, miR-150 is important in T cell development; the upregulation of miR-150 inhibits the expression of the target gene NOTCH3^27^.

## MiRNA is required for T cell activation, proliferation, and apoptosis

The activation of T cells depends on TCR and co-stimulatory molecules, such as CD28. T cells proliferate vigorously upon productive activation, leading to clonal expansion. Many miRNAs are involved in the regulation of T cell proliferation. After an external stimulation is detected, T cells greatly increase the expression of miR-214 and inhibit the expression of the target gene phosphatase and tensin homolog, which is deleted in chromosome ten (PTEN); as a result, T cell proliferation is enhanced^18^. In CD8^+^T cell activation, miR-130 and miR-301 are upregulated, resulting in a decreased CD69 expression through two miRNAs binding to the 3′-UTR of mRNA of CD69^28^. Grigorev *et al.*^29^ demonstrated that high miR-155 and miR-221 expressions inhibit the expression of PIK3R1 as a co-target gene and further inhibits cell proliferation and cytokine production in CD4^+^ T cells. A highly expressed miR-182 binds to Foxo1 of the 3′-UTR of mRNA and inhibits protein synthesis; this process results in an enhanced proliferation of T helper cells^30^. The function of miR-21 depends on the status of T cells. For instance, miR-21 regulates the survival of activated memory T cells and induces CCR-7 expression in activated naive T cells^31^.

The transcription factor c-Myc regulates cell proliferation, growth, and apoptosis. c-Myc also activates the expression of the miR-17~92 gene cluster. c-Myc is directly bound to this locus as observed in the results of chromatin immunoprecipitation assay. Two members of the miR-17~92 gene cluster, specifically miR-17-5p and miR-20a, negatively regulate the expression of E2F1, a known downstream gene of c-Myc. These findings have revealed a new mechanism by which c-Myc promotes E2F1 transcription and simultaneously limits translation; thus, cell proliferation is accurately controlled^32^. In Th1 cells, miR-19 and miR-17, as members of miR-17~92 gene cluster, target PTEN and cAMP-response element binding protein (CREB1), respectively; these miRNAs also participate in the immune response of Th1 cells by enhancing T cell proliferation, promoting cytokine production, and inhibiting apoptosis^33^^,^^34^. As CD8^+^T cells are stimulated by viral infection, miRNA profiling is instantaneously changed. Among these miRNAs, miR-17~92 cluster fails to regulate the expression levels of proteins with controlled effector and memory CD8^+^T cell differentiation^35^. In mice, high miR-17 to 92 expression in lymphocytes causes lymphoproliferative disease and autoimmunity; as a result, the mice died prematurely. Lymphocytes from these mice are also hyperpoliferative and resistant to apoptosis possibly because miR-17~92 suppresses the expression of the tumor suppressor PTEN and the proapoptotic protein Bim^36^. Another study has shown that miR-150 and miR-139 regulate perforin, eomesodermin, and IL-2Rα expression in the progression of CTL cell differentiation, which is involved in inflammation and antigen stimulation^37^.

Many miRNAs are involved in the regulation of T cell apoptosis. In CD4^+^ T cells from patients with relapsing forms of multiple sclerosis, miR-15a and miR-16-1 are downregulated and the target-gene B-cell lymphoma 2 (Bcl-2) was upregulated; this result affects the progression of apoptosis^38^. Bim is a member of Bcl-2 family and participates in the mediation of lymphocyte apoptosis^39^. In patients with malignant lymphoma, glucocorticoid inhibits the expression of miR-17~92 gene cluster and this result is consistent with a high Bim expression. miR-17~92 overexpression reduces Bim expression levels and inhibits glucocorticoid-mediated apoptosis. By contrast, the knockdown of miR-17~92 increases Bim protein expression, thereby enhancing apoptosis^40^. However, the function of these miRNAs in T cells remains unclear. In another study, miR-122 is expressed in cutaneous T cell lymphoma (CTCL). In apoptotic CTCL cells triggered by various chemotherapeutic drugs, miR-122 is further upregulated. MiR-122 overexpression induces Akt activation and p53 inhibition, resulting in the resistance to chemotherapy-induced apoptosis. These data indicated that miR-122 amplifies the Akt/p53 anti-apoptosis pathway^41^.

## MiRNA functions in the development of functional peripheral T cell subsets

Mature peripheral T cells consist mainly of regulatory T cells (Tregs), CD4, and CD8 T cells. These cells further differentiate into various functional subsets. The function of miRNAs in the development of divergent T cell subsets has been elucidated in several studies.

The miRNA expression in naive, effector, and memory CD8^+^ T cells has been studied and dynamic changes in the miRNA profile during peripheral T cell differentiation have been revealed. The downregulation of miRNAs has been observed in effector T cells compared with naive cells and memory T cells. In effector T cells, six miRNAs (let-7f, miR-15b, miR-142-5p, miR-150, miR-142-3p, and miR-16) are expressed at a low extent^8^. By contrast, a few miRNAs, such as miR-21, are highly expressed in effector T cells compared with memory and naive T cells. In antigen-stimulated CD8^+^T cells, miR-155, miR-21, and miR-146a are upregulated^14^.

Using an *in vitro* system in which activated CD8 T cells are driven by IL-2 or IL-15 function as either effector memory cells or central memory cells, we observed that numerous miRNAs, such as miR-150, miR-155, and the let-7 family, are associated with the development of these memory T cell subsets. In particular, miR-150 regulates the protein expression of Kv channel interacting protein 1 (KChiP1) in mouse central memory T cells^42^. These findings demonstrated the possible functions of these miRNAs in the further development of peripheral T cells.

T helper cells can be divided into Th1, Th2, Th17, and Th9 cells based on cytokine profiles; miRNAs are important in the differentiation and function of these T cell subsets. For instance, miR-142-5p is associated with CD4^+^CD25^+^ T cell proliferation by binding to the 3′-UTR of the mRNA of GARP^43^. MiR-21 regulates Th1 and Th2 polarization and inflammatory response via the IL-2 and IFN-γ signaling pathways^44^. The targeted ablation of miR-21 in mice results in reduced lung eosinophilia after allergen challenge, thereby significantly increasing Th1 cytokine IFN-γ levels and IL-12 production by dendritic cells. Mice infected with *Listeria monocytogenes* or *Mycobacterium bovis* bacillus Calmette-Guérin (BCG) exhibit a downregulated miR-29 expression in IFN-γ-producing natural killer cells, CD4^+^T cells, and CD8^+^T cells. MiR-29 suppresses IFN-γ production by directly targeting IFN-γ, T-bet, and Eomes mRNA^45^^,^^46^. IL-23 also participates in Th17 responses. One study showed that miRNA let-7f inhibits the expression of IL-23 reporter in CD4^+^T cells^47^; this result indicated the function of these miRNAs in Th17 responses. MiR-125p is transfected into naive T cells, which terminate differentiation from naive T cells to effector cells^48^.

Treg cells are responsible for the induction of immune tolerance and immune homeostasis. In the expression profile of miRNA from Treg cells, miR-24, miR-210, miR-95, and miR-145 are upregulated; by comparison, miR-24 and miR-210 negatively regulate FOXP3; miR-95 positively regulates FOXP3 via an indirect mechanism. In addition, miR-145 negatively regulates CTLA-4 expression^49^. Takahshi *et al.*^50^ reported that miR-10a is highly expressed in Treg cells. MiR-10a is induced by retinoic acid and transforming growth factor-β (TGF-β), which target the transcriptional suppressor Bcl-6 and the co-suppressor Ncor2; as a result, the phenotypic conversion of Treg into follicular Th cells is decreased. Moreover, miR-10a limits the generation of Th17 cells from the differentiation of Treg cells50. In stimulated Treg cells, miR-155 is upregulated; FOXOa3, the target of miR-155, negatively affects the Akt signaling pathway^51^. MiR-146 also inhibits the signal transducer and activator of transcription 1 (STAT1) expression by controlling Th1 responses via Treg cell-mediated regulation^52^.

## MiRNA in cancer immunity

Type-1 T cells are important for the effective inhibition of tumor immune responses. In immune microenvironments, immune cells interact with cancer cells; with the passage of time, immune cells are stimulated by cancer cells and act against cancer cells by secreting small molecules. MiRNAs are a large family of small regulatory RNAs that function at a post-transcriptional level regulated by different processes of cell functions, including immune system regulation. miR-17~92 are downregulated in tumor microenvironments with specific T cells compared with normal T cells^53^. MiRNAs also regulate the co-stimulation of expressed molecules, such as intercellular adhesion molecule-1 (ICAM-1)^54^, B7-H1^55^, B7-H3^56^, and cytokine^57^, which co-exist in tumor microenvironments. MiRNAs affect anti-tumor immunity by balancing the development, differentiation, and function of immune cells as well as the secretion of cytokines in local tumor microenvironments (**Table 1**).

**Table 1:** Mechanism of miRNA-regulated T cells

Stage	MiRNAs	Cell expression and mechanism	Reference
Development	MiR-125b	MiR-125b is involved in hematopoietic stem cell survival in lymphoid balance	Ooi *et al.*^21^
	MiR-181a	MiR-181a enhances DP cell antigen sensitivity, and inhibits Bcl-2, CD69 expression, and TCR effects the positive clone selection of T cells	Wu *et al.*^8^
			Liu *et al.*^22^
			Li *et al.*^25^
	MiR-150	During T cell maturation process, upregulation miR-150 inhibits the expression of the target gene NOTCH3	Ghisi *et al.*^27^
Activation Proliferation Apoptosis	MiR-214	After stimulation, highly expressed miR-214 inhibited target gene PTEN expression in the proliferation of T cells	Jindra *et al.*^18^
	MiR-130/301	During CD8^+^T cell activation, miR-130 and MiR-301 are upregulated, inhibit of CD69 expression	Zhang *et al.*^28^
	MiR-155 MiR-221	High expressions of miR-155 and miR-221 inhibited the expression of PIK3R1 in CD4^+^T cells	Grigoryev *et al.*^29^
	MiR-182	MiR-182 binds to 3′-UTR of mRNA of Foxo1 in T helper cells	Stittrich *et al.*^30^
	MiR-21	MiR-21 regulates the survival of activated memory T cell and induces CCR-7 expression in activated naive T cells	Smigielska-Czepiel *et al.*^31^
	MiR-17~92	In Th1 cells, miR-19 and miR17 target PTEN and CREB1, respectively, are involved in Th1 cell immune response. MiR-17~92 gene cluster exhibits reduced expression and inhibits Bim and PTEN expressions, which affect apoptosis	Jiang *et al.*^33^
			Xiao *et al.*^36^
	MiR-150	MiR-150 and miR-139 regulate perforin, eomesodermin, and IL-2Rα expressions in differentiation CTL cell, involved in inflammation and antigen stimulation	Trifari *et al.*^37^
	MiR-139		
	MiR-15a/16-1	MiR-15a/16-1 is downregulated, the upregulated target gene Bcl-2 affects the progression of apoptosis in CD4^+^T cells	Lorenzi *et al.*^38^
	MiR-17~92	MiR-17~92 gene cluster inhibits Bim expression, which facilitates glucocorticoid-induced apoptosis in CD4^+^T cells	Molitoris *et al.*^40^
	MiR-122	In apoptosis CTCL cells, miR-122 is upregulated, which activates Akt-related pathway; indicated that miR-122 amplifies the Akt/p53 anti-apoptosis pathway	Manfè *et al.*^41^
The development of functional peripheral T cell subset	MiR-150	In IL-15/IL-2-induced CD8 T cells, the target gene of miR-150 is KChiP1, which affects cell phenotype and self-renewal capacity via a negative feedback pathway	Almanza *et al.*42
	MiR-142-5p	MiR-142-5p associated with CD4+CD25+ T cell proliferation by binding to the 3′-UTR of mRNA of GARP	Zhou *et al.*43
	MiR-29	MiR-29 regulates T-box transcription factors and IFN-γ production in Th cells	Ma *et al.*45 Steiner *et al.*46
	MiR-125p	MiR-125p terminates the differentiation from naïve T cells to effector cells	Rossi *et al*.48
	MiR-24 MiR-210 MiR-145	MiR-24, miR-210, miR-95, and miR-145 are upregulated, miR-24 and miR-210 miR-95 regulate FOXP3 expression miR-145 negatively regulates the CTLA-4 expression in Treg cells	Fayyad-Kazan *et al*.49
	MiR-10a	MiR-10a restricted Treg cell differentiation into Th17 cells	Takahashi *et al.*50
	MiR-155	MiR-155 is upregulated, FOXOa3, the target gene of miR-155, negatively affect the Akt signaling pathway in stimulated Treg cells	Yamamoto *et al.*51
	MiR-146	MiR-146 inhibits STAT1 expression by controlling Th1 response via Treg cell-mediated regulation	Lu *et al.*52
Cancer immunity	MiR-222 MiR-339	Dicer-regulated miR-222 and miR-339 promote resistance of cancer cells to cytotoxic T-lymphocytes by downregulating of ICAM-1	Ueda *et al.*54
	MiR-513	Cryptosporidium parvum induces B7-H1 expression in cholangiocytes by downregulating miR-513	Gong *et al.*55
	MiR-29	MiR-29 modulates the expression of the immunoinhibitory molecule B7-H3	Xu *et al.*56
	MiR-122	MiR-122 binds the IL-1α 3’-untranslated region	Gao *et al.*57

## Conclusion

Increasing evidence suggests that miRNAs are important in the progression, development, and formation of immune systems. Therefore, miRNA regulatory networks should be further investigated in the context of disease settings to help elucidate the function of miRNAs in tumor microenvironments and inflammatory environments. Studies have focused on the mechanism of miRNA regulation. MiRNA should be engineered and applied in tumor microenvironments to inhibit oncogenes or suppress gene expression. In this way, miRNAs can function more effectively and accurately. Understanding the mechanism of T cell regulation by miRNAs, we may develop new therapies. Studies on engineering miRNAs have provided valuable information regarding the methods by which we could improve anti-tumor activity against solid tumors, as well as immune, autoimmune, and lymphatic diseases.
